# Job stress among community health workers: a multi-method study from Pakistan

**DOI:** 10.1186/1752-4458-2-15

**Published:** 2008-10-28

**Authors:** Zaeem Haq, Zafar Iqbal, Atif Rahman

**Affiliations:** 1 Pakistan Initiative for Mothers and Newborns (PAIMAN) House 6, Street 5, F-8/3, Islamabad, Pakistan; 2 Human Development Research Foundation, Pakistan; 3 University of Liverpool, Liverpool, UK

## Abstract

**Background:**

In low income countries, the task of providing primary health care is often the responsibility of community health workers. In Pakistan, community workers called Lady Health Workers (LHW) deliver basic health care at the doorstep in the rural areas and urban slums. Evaluations show that it is a successful programme but point out inconsistencies in the quality of service provided. In order achieve this, it would be important to obtain the workers' viewpoint on their job-description, the problems they face and the levels of stress they encounter.

**Methods:**

We conducted a multi-method study to investigate the aforementioned issues. All LHWs from one typical rural sub-district in Rawalpindi were surveyed. Focus group discussions with a sub-set of these workers were also conducted.

**Results:**

About a quarter of the LHWs were found to have significant occupational stress. Factors associated with stress included having low socio-economic status and having to travel long distances for work. Inconsistent medical supplies, inadequate stipends, lack of career structure and not being equipped to communicate effectively with families were the main factors for job dissatisfaction among these workers.

**Recommendations:**

Improvement in remuneration, better administration of supplies and a structured career path should be ensured for better performance of community health workers. In addition, communication skills learning should be an essential part of their training programme.

## Background

Health workers deployed from within their own communities to deliver basic health care have various titles but "Community Health Worker (CHW)" is the term most commonly used to describe this cadre. According to the WHO: "Community Health Workers should be members of the communities where they work, should be selected by the communities, should be answerable to the communities for their activities, should be supported by the health system but not necessarily a part of its organization, and have shorter training than professional workers." These workers play a crucial role in improving access and broadening coverage of health services in far flung areas and can contribute to improved health outcomes [1].

The "Lady Health Worker" (LHW) of the National Programme for Family Planning and Primary Health Care in Pakistan broadly fits into the definition of community health worker, and is a crucial component of the health care delivery system of the country. The Lady Health Workers Programme (LHWP) is a federally funded development programme working at the grass root level since 1994. About 96,000 workers and their supervisors have been trained and deployed in all the 135 districts of Pakistan. They currently cover about 65% of the target population (rural and urban slums), and full coverage is planned in the next few years [2]. Pakistan has high maternal and infant mortality [3], low women's access to health services [4,5] with less than one third going to health centres unescorted [6], and low contraceptive usage among the rural population [7,8]. Thus the job of the LHW is challenging.

The job description of the LHW has evolved over time. Initially it included health education and basic preventive services for family planning; maternal and child health; improving nutrition; basic hygiene and sanitation; and child immunization. Today it also includes mass immunization for polio eradication; newborn care; maternal immunization with tetanus toxoid (TT); referral of eligible cases to health facilities and regular record-keeping for updating the management information system (MIS) of the programme; community management of tuberculosis; and health education on HIV-AIDS and Hepatitis [2]. Lady Health Workers are seldom consulted when their job description changes. This ever-enlarging scope of work of the LHW in which they have little say can result in occupational stress. This condition defined as "any physical or psychological event perceived as potentially constituting physical harm or emotional distress", if present in health workers can have an adverse impact on their efficiency [9-11].

Since the commencement of LHWP in Pakistan, a number of programme evaluations have been carried out. Most extensive among these was the national-level, external evaluation conducted during 2000–2001 [12]. It concluded that the programme succeeded in creating a large sized organization comprising female community health workers and establishing a functional programme management and supply system. It found evidence that the programme improved the uptake of important health services in areas covered by its LHW. At the same time it recommended that the quality of work needed improvement. But there was no information on the LHWs' own views about their job description and levels of occupational stress. These factors would be important in the improvement in the quality of service delivery and under performance/utilization of existing LHWs. The domain in general is poorly researched and systematic reviews have pointed out knowledge gaps in areas like job satisfaction/dissatisfaction and job retention/attrition [13,14].

We assessed the perceived level of job stress, personal efficiency and quality of service delivery by LHW. An attempt was made to understand factors which might be responsible for below optimal performance of LHWs.

## Methods

### Study Area

The study was conducted in Tehsil Kahuta, one of the six Tehsils (sub-districts) of District Rawalpindi, located 60 Km southeast of the capital city of Islamabad. Kahuta has an area of 1096 Sq. Kms, a population of 313,200, and consists of 20 union councils (smallest administrative unit, each consisting of 5–12 villages). The Tehsil is a typical rural area of Pakistan, and is similar to most of the under-developed rural areas in South Asia. The average household consists of 6.2 members. Most families depend on subsistence farming, supplemented by earnings of one or more of the adult male members serving in the public sector, the armed forces or the private sector. Male and female literacy rates are 80% and 50% respectively. The infant mortality rate is 84 per 1000 live births. There are 20 basic health units and two rural health centres, employing 28 doctors, 12 midwives, 15 vaccinators, and about 200 LHWs, providing basic primary health care.

### Subjects and Study design

Multi-stage, stratified, random sampling was done. 15 out of 20 union councils of the sub-district Kahuta were randomly selected. Union councils not covered by LHW were excluded. The sample comprised all LHWs from the selected union councils who were physically healthy (without any diagnosed or known chronic illness), aged 18–50 years, based at their respective villages, married or un-married, had education of grade 8 and above, willing to participate in the study and having at least one year of work experience as LHW.

The study design was cross sectional. It consisted of three sub-components (i) survey type investigation aimed at describing accurately the characteristics of the LHW specific beliefs, attitude and opinion related variables including socio-demographic profile (ii) comparison of naturally occurring variables of work-related stress (iii) qualitative analysis of LHWs work-related problems and their suggestions to improve their role as primary agent for primary health care. The study used three techniques for the process of data collection including questionnaire, interview and focus group discussion.

### Instruments

Two experienced Research Assistants (RAs) interviewed the LHWs individually at their homes. To collect data for the development of *'socio-demographic profile of the LHW' *a form was designed by adopting multiple methods including key informant interviews, and consultations with professional researchers/experts in the field. It was pre-tested and finalized before the actual data collection process. *'Assessment of mental distress and work-related stress' *was done by using the Self-Reporting Questionnaire, briefly known as SRQ-20 [15]. The SRQ has been translated, adapted and validated as a culturally sensitive and effective instrument in Pakistan [11,16].

Further, to supplement the SRQ-20, two additional scales, namely sources of 'job pressure' and 'job satisfaction' were used. The questionnaire was provided and the LHW was asked to mark her responses on a scale of 1 to 5 with reference to her sources of job pressure and job satisfaction. These two scales were translated, culturally adapted and pre-tested using a standard procedure [17]. Originally, Occupational Stress Indicators (OSInd) was developed by Cooper et al [18].

To document '*Perceived job description & identification of LHWs problems*' a form containing two open-ended questions was used. The responses to these questions were recorded on a proforma for further content analysis. In order to conduct a *Qualitative analysis of LHWs problems & suggestions *focus group discussions (FGDs) were organized within the field area. A sub-sample of LHWs participated in the FGDs.

### Data collection and analysis

A total of 150 LHWs were randomly selected according to inclusion criteria and asked to fill out the questionnaires. Interviews were also conducted with all these LHWs. Six Focus Group Discussions (FGDs) were organized using a sub-sample (48/150) of these LHWs. The RAs obtained lists of all working LHWs in the selected union councils from the concerned authorities. The RAs collected data from the LHWs after getting their informed consent. All the data including socio-demographic variables, SRQ, LHWs perceptions about their job description and problems, and sources of job pressure and job satisfaction scales was collected in a single visit.

Six focus groups were organized with subgroups of LHWs for the assessment of their work-related problems and practical suggestions to improve their job related efficiency/quality. The collected data were coded and entered in a computerized database by an expert data controller/statistician. A trained statistician performed the data analysis and generated outputs under the supervision of the principal investigator.

The quantitative data was analyzed using the Statistical Package for Social Sciences (SPSS 10.0), applying '*inferential*' t-test and descriptive statistical methods as appropriate. Inductive content analysis method was used to analyze the qualitative data of the focus group discussions to generate relevant result categories.

The study was completed between15 April 2003 and 31 January 2004. Ethics approval was obtained from the Human Development Research Foundation (HDRF), Pakistan.

## Results

All 150 interviews were successfully completed. Table 1 shows the sociodemographic characteristics of the sample. Table 2 presents a summary of SRQ, SPJ and OSI scores. In SRQ, a cut-off score of 8 or more was taken to indicate the presence of mental distress. Based on this cut-off, 26% of respondents had mental distress. Taking a score of 40–50 (1 sd) on the SPJ as a cut off revealed that 14% of the respondent LHWs were experiencing high job pressure, while taking > 51 (2 sd) as the cut-off point showed that an additional 5% had a very high job pressure, resulting in a cumulative figure of 19% of respondents having significant job pressure.

**Table 1:** Socio-demographic variables (N = 150)

**Demographic Variable**	**Mean**	**Standard Deviation**	**Range**
1. Age (Years)	34.3	7.2	19 – 50
2. Education (Grade)	10	2	8–12
3. Number of Children	3	2	0 – 8
4. Number of Family Members	7	3	2 – 18
5. Duration of Job (Years)	4.1	2.8	1 – 9.1
6. Daily Travel (Km)	2.7	2.0	0.5 – 6.0
7. Distance from BHU (Km)	4.1	3.8	0.5 – 20.0
8. Monthly Family Income (PKR)	4,790	3,293	1,580 – 30,000

**Table 2:** Summary table of SRQ, SPJ and OSI scores

**N = 150**	**SRQ**	**SPJ**	**OSI**
Mean	5.1	29.0	34.0
Std. Deviation	4.5	11.3	8.1
Range	19	52.0	40.0
Minimum	0	15.0	10.0
Maximum	19	67.0	50.0

SRQ = Self-Reporting Questionnaire, to assess work related mental distress

SPJ = Sources of Pressure on Job, to identify specific sources of pressure on job

OSI = Occupational Stress Indicators, to assess level of job satisfaction

Comparison was made with respect to different socio-economic variables given in Table 1. Three variables had a statistically significant effect on the LHW resulting in job stress. These included having to travel 2 km or more every day to perform her job, living a distance of 3 km or more away from the BHU, and having a family income of PKR 4,000 per month or less. Age, marital status, number of children, and living in joint or nuclear family had no impact on the psychological state and did not appear to be a factor for increased job pressure.

Factors leading to increased job pressure were also explored. Overall, the absence of career advancement was a source of moderate to very high pressure in 53% of the respondents (Table 3) while working with opposite sex was causing moderate to very high pressure in 23% of respondents. Being undervalued by the department was having a similar effect on 23% of the respondent LHWs while 29% of them felt moderate to high pressure because of the things not under their direct control.

**Table 3:** Sources of pressure in job for LHWs (item-level responses on SPJ)

No	**Categories**	**Very Little**	**Little**	**Moderate**	**Much**	**Most**
1	Absence of potential career advancement	52 (34%)	19 (12%)	24 (16%)	25 (17%)	30 (20%)
2	Working with the opposite sex	84 (56%)	31 (20%)	12 (8%)	8 (5%)	15 (10%)
3	Factors not under direct control	78 (52%)	26 (17%)	22 (14%)	14 (9%)	10 (6%)
4	Being undervalued by the department	81 (54%)	32 (21%)	16 (10%)	14 (9%)	7 (4%)

Occupational Stress Indicator (OSI) was used as a measure of job satisfaction among the respondents and the mean OSI for the group was 33.96 (Table 2). On a scale of 1–5, the level of job satisfaction and factors responsible were also explored. Overall, 56% of the respondents expressed very little to moderate job satisfaction. 79% reported very little to moderate satisfaction with amount of salary, 39% of the respondents reported low satisfaction with the professional skills they had while 65% had a low level of inspiration in terms of professional development (Table 4).

**Table 4:** Job-satisfaction among LHWs

No	Categories	Very Little	Little	Moderate	Much	Most
1	Level of job satisfaction	15 (10%)	12 (8%)	58 (38%)	31 (20%)	34 (22%)
2	Satisfaction with the amount of work	20 (13%)	16 (10%)	32 (21%)	43 (28%)	39 (26%)
3	Satisfaction with amount of salary	65 (43%)	26 (17%)	29 (19%)	14 (9%)	16 (10%)
4	Satisfaction with professional skills	10 (6%)	5 (3%)	45 (30%)	35 (23%)	55 (36%)

**Figure 1:**
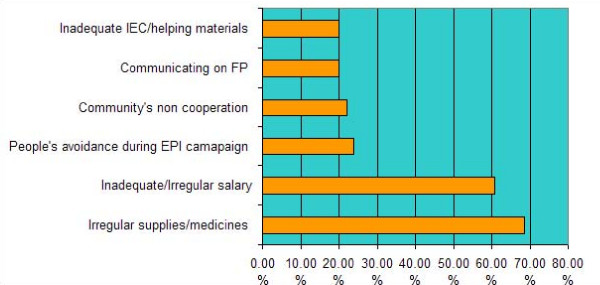
Most common problems reported by the LHWs.

The researchers also asked an open-ended question about the main problem the LHW faced while performing her job. Problems that were reported with high frequency (20% or more) have been shown in Figure 1. The most common problem reported was dealing with administrative inefficiency such as irregular supply of medicines and vaccines (70%) and not getting their salary on time. Inadequate salary was the next biggest problems reported by over 60% of the respondents. Other problems included difficulty motivating mothers and families to get their children immunized and take preventive measures, difficulty in communicating on family planning issues, non cooperative attitudes of community and inadequate information, education, communication (IEC) materials and other job aids.

## Discussion

To our knowledge, this is the first study from Pakistan that focused on the LHW's work related stress and various factors contributing to it. It has clearly shown that over a quarter (26%) of the LHWs of the National Programme are mentally distressed. In addition to the administrative issues like inadequate amount and irregular disbursement of salary, and inadequate and irregular supply of medicine and other supplies, the rest of the 'causes for concern' are related to communication and interpersonal skills. The study has also established that a large proportion of health workers of this important public health program are not satisfied with their job because of the perceived absence of professional development and lack of career path.

A motivated workforce has been described as central to any health system. The improvement of human resource management in the health sector has been recommended in order to achieve millennium development goals (MDGs) [19]. The third party evaluation of LHW Programme [12] also mentioned the need to improve quality of the work through improved management of the workforce. To achieve this, LHW's own opinion about the factors contributing to their job satisfaction/dissatisfaction must be studied. The qualitative nature of our study has enabled us to gain some insights in this area. At the same time, this qualitative study conducted in one sub-district has obvious limitations and is only suggestive at best.

The potential role of CHWs in improving community health has been acknowledged especially in resource poor countries. Haines et al [14] have described that owing to the inverse relationship of density of health workers (doctors, nurses, midwives) with maternal, infant and under 5 mortality; coupled with high cost of training doctors and nurses and the low use of services based in health facilities in many areas, there is a possibility to make substantial health gains from the use of community health workers. The Task Force for Scaling up Education and Training for Health Workers [20] recommended improving education of these workers through quality assurance programs and urged international action to scale up the production of quality health workers.

Other studies have also reported areas for improvement in the structure and performance of CHW programs including the LHWP of Pakistan. The low salary and lack of career path was highlighted by Afsar et al [21] as a reason for job dissatisfaction among the LHWs. Mumtaz et al. [22] reported abusive hierarchical management structure, disrespect from male colleagues, lack of sensitivity to women's gender-based cultural constraints, conflict between domestic and work responsibility and poor infrastructural support as the important problems faced by female primary health care workers from their study conducted in 1998 when the program was only four years old. Our study suggests that the disrespect from male colleagues and conflict between domestic and work related responsibility has improved while the other factors remain the same.

Douthwaite & Ward [23] found that the LHWP succeeded in increasing the use of modern contraceptives by rural women. According to them women served by LHW were significantly more likely to use a modern reversible methods than women in communities not served by LHW after controlling for various individual and household characteristics. They advocated for continuation of providing doorstep services through community-based workers to achieve universal access to safe family planning methods. Our study suggests that communication on family planning is still perceived as a difficult area by these workers and, while the programme should be continued, some interpersonal communication (IPC) capacity building measures are needed to further improve performance and outcomes.

Multifaceted interventions (e.g. training plus supervision) which address multiple determinants of performance have been recommended [13] to improve CHW performance. We add that improvement in remuneration; clear career path and improved administration are also required. In addition, empowering communication techniques should be built into the training and on-going supervision processes to improve the effectiveness of the community health workers.

## Competing interests

The authors declare that they have no competing interests.

## Authors' contributions

ZH carried out analysis of data, conceptualized this paper and drafted the manuscript while ZI and AR helped in the design and supervision of the study and reviewed drafts. All authors read and approved the final manuscript.

## References

[B1] World Health Organization (2007). Community health workers: What do we know about them?.

[B2] Ministry of Health Government of Pakistan (2008). Internal assessment of Lady Health Workers' Programme 2007.

[B3] UNICEF (2007). The State of the World's Children 2007.

[B4] Khan A (1999). Mobility of women and access to health and family planning services in Pakistan. Reproductive Health Matters.

[B5] Fikree F, Khan A, Kadir MM, Sajan F, Rahbar M (2001). What influences contraceptive use among young women in urban squatter settlements of Karachi Pakistan?. International Family Planning Perspectives.

[B6] Sathar Z, Kazi S (1997). Women's autonomy, livelihood and fertility: a study of rural Punjab.

[B7] Cernada GP, Rob AKU, Ameen S, Ahmed MS (1993). A situation analysis of family welfare centres.

[B8] Population Council (1997). The gap between reproductive intentions and behaviour: a study of Punjabi men and women.

[B9] Clegg A (2001). Occupational stress in nursing: a review of the literature. Journal of Nursing Management.

[B10] Evans L (2002). An exploration of district nurses' perception of occupational stress. British Journal of Nursing.

[B11] Delvaus N, Razavi D, Farvacques C (1998). Cancer Care-a stress for health professionals. Soc Sci Med.

[B12] Oxford Policy Management (2002). External evaluation of the National Programme for Family Planning and Primary Health Care: Summary of Final Report.

[B13] Rowe A, Savigny D, Lanata C (2005). How can we achieve and maintain high-quality performance of health workers in low-resource settings?. Lancet.

[B14] Haines A, Sanders D, Lehmann U (2007). Achieving child survival goals: potential contribution of community health workers. Lancet.

[B15] World Health Organization (1994). A User's Guide to the Self -Reporting Questionnaire (SRQ).

[B16] Minhas FA, Iqbal K, Mubbashar MH (1995). Validation of self-reporting questionnaire in primary care setting of Pakistan. Journal of Pakistan Medical Association.

[B17] Rahman A, Iqbal Z, Waheed W, Husain N (2003). Translation and cultural adaptation of health questionnaires. J Pak Med Assoc.

[B18] Cooper C, Sloan S, Williams S (1988). The occupational stress indicators (OSI).

[B19] Hongoro C, McPake B (2004). How to bridge the gap in human resources for health. Lancet.

[B20] Crisp N, Gawanas B, Imogen S (2008). Training the health workforce: scaling up, saving lives. Lancet.

[B21] Afsar H, Younus M (2005). Recommendations to strengthen the role of lady health workers in the National Program for family Planning and Primary Health Care in Pakistan: the health workers perspective. Journal of Ayub Medical College.

[B22] Mumtaz Z, Salway S, Waseem M (2003). Gender-based barriers to primary care provision in Pakistan: the experience of female providers. Health Policy and Planning.

[B23] Douthwaite M, Ward P (2005). Increasing contraceptive use in rural Pakistan: an evaluation of the Lady Health Worker Programme. Health Policy and Planning.

